# Longitudinal analysis of quality of life following treatment with Asunercept plus reirradiation versus reirradiation in progressive glioblastoma patients

**DOI:** 10.1007/s11060-019-03320-x

**Published:** 2019-11-02

**Authors:** Wolfgang Wick, Andriy Krendyukov, Klaus Junge, Thomas Höger, Harald Fricke

**Affiliations:** 1 Department of Neurology and Neurooncology Program, National Center for Tumor Diseases, Heidelberg University Hospital, Heidelberg, Germany; 2 Apogenix AG, Heidelberg, Germany; 3 Scope International AG, Mannheim, Germany

**Keywords:** Asunercept, Recurrent glioblastoma, Quality of life, Time to deterioration

## Abstract

**Purpose:**

Glioblastoma is an aggressive malignant cancer of the central nervous system, with disease progression associated with deterioration of neurocognitive function and quality of life (QoL). As such, maintenance of QoL is an important treatment goal. This analysis presents time to deterioration (TtD) of QoL in patients with recurrent glioblastoma receiving Asunercept plus reirradiation (rRT) or rRT alone.

**Methods:**

Data from patients with a baseline and ≥ 1 post-baseline QoL assessment were included in this analysis. TtD was defined as the time from randomisation to the first deterioration in the EORTC QLQ-C15, PAL EORTC QLQ-BN20 and Medical Research Council (MRC)-Neurological status. Deterioration was defined as a decrease of ≥ 10 points from baseline in the QLQ-C15 PAL overall QoL and functioning scales, an increase of ≥ 10 points from baseline in the QLQ-C15 PAL fatigue scale and the QLQ-BN20 total sum of score, and a rating of “Worse” in the MRC-Neurological status. Patients without a deterioration were censored at the last QoL assessment. Kaplan–Meier estimates were used to describe TtD and treatment groups (Asunercept + rRT or rRT alone) were compared using the log-rank test.

**Results:**

Treatment with Asunercept + rRT was associated with significant improvement of TtD compared with rRT alone for QLQ-CL15 PAL overall QoL and physical functioning, and MRC Neurological Status (p ≤ 0.05). In the Asunercept + rRT group, QoL was maintained beyond progresison of disease (PoD).

**Conclusion:**

Treatment with Asunercept plus rRT significantly prolongs TtD and maintains QoL versus rRT alone in recurrent glioblastoma patients.

## Introduction

Glioblastoma (GB) is the most aggressive malignant cancer of the central nervous system and accounts for > 60% of adult brain tumours [1]. Median survival from time of GB diagnosis is 14–15 months [2, 3], and disease progression is often associated with a gradual deterioration of neurocognitive function, quality of life (QoL) and functional independence [4]. No treatment standard exists for GB at progression, but available therapeutic strategies include reoperation, reirradiation (rRT), alkylating chemotherapy with temozolomide or nitrosoureas (such as lomustine), bevacizumab, and experimental agents used within clinical trials [5–9]. With the absence of standard therapy, enrolment into clinical trials is recommended by guidelines as the preferred treatment approach [6–8, 10, 11]. This highlights the urgent need for new innovative approaches for the treatment of recurrent GB (rGB).

Activation of the CD95 (Fas)/CD95L (Fas ligand) signaling pathway plays an important role in invasive growth and migration in GB [5, 12–15]. Asunercept/APG 101 is a recombinant glycosylated fusion protein that consists of the extracellular domain of human CD95 linked to the Fc domain of human IgG1. It was designed to selectively bind to CD95L and thereby disrupt CD95/CD95L interaction. The scientific rationale for Asunercept in recurrent glioblastoma is supported by a number of in vitro and in vivo nonclinical studies that show its enhanced effect when administered in combination with radiotherapy [13, 15]. A Phase II clinical trial (NCT01071837) aimed to assess the combination of Asunercept with rRT to support the rationale that Asunercept enhances the efficacy of rRT [13, 15]. There is evidence that RT temporarily disrupts the blood–brain-barrier [16] and thus may facilitate Asunercept entering the tumour. The study demonstrated improved 6-month progression-free survival (PFS-6) for Asunercept + rRT (20.7% [95% confidence interval: 11.2–33.4]) compared with rRT alone (3.8% [95% confidence interval: 0.1–19.6]) [5].

The burden of disease in patients with GB is high [17, 18] and has a significant impact on QoL, including sleep disruption, inability to concentrate, depression, financial difficulties, and impaired professional, personal, and social lives [19]. Given the poor prognosis of GB and rGB with currently available treatment options, maintenance of QoL is an important therapeutic goal [6, 7]. Beyond progression-free survival (PFS) and overall survival (OS), maintenance or even improvement of QoL is an important goal of treatment. The current analysis presents time to deterioration (TtD) of QoL in patients with rGB receiving Asunercept + rRT, compared with those receiving rRT alone.

## Methods

This Phase II study (NCT01071837) followed a Simon two-stage design. Methods and primary and secondary outcome results have been previously published [5]. In brief, a randomised control arm with rRT alone was added to avoid under- or overestimation of a signal from Asunercept [5]. Patients (N = 91) with GB at first or second progression were randomised 1:2 between rRT alone (36 Gy; five times 2 Gy per week) or rRT + Asunercept (400 mg weekly as a 30-minute i.v. infusion) [5]. Seven patients dropped out without receiving study treatment, leaving 84 patients for the Full Analysis Set (FAS). All procedures performed were in accordance with the ethical standards of the institutional and national research committees and with the 1964 Helsinki declaration and its later amendments or comparable ethical standards [5]. All patients were required to give signed informed consent before enrolment.

The current post-hoc analysis assessed TtD of QoL using data from this study. QoL was assessed at baseline and every 6 weeks after the end of rRT until the end of the study, not including follow-up periods. Patients with a baseline and ≥ 1 post-baseline QoL assessment were included. TtD was defined as the time from randomisation to the first deterioration in EORTC QLQ-C15 PAL, EORTC QLQ-BN20 and Medical Research Council (MRC)-Neurological status. Deterioration was defined as a decrease of ≥ 10 points from baseline in the QLQ-C15 PAL overall QoL and functioning scales, an increase of ≥ 10 points from baseline in the QLQ-C15 PAL fatigue scale and the QLQ-BN20 total sum score, and a rating of “Worse” in the MRC-Neurological status. Patients without deterioration were censored at the last QoL assessment [20, 21]. Kaplan–Meier estimates were used to describe TtD and both treatment groups were compared using the log-rank test. The relationship between time to progression of disease (TtPoD) and QoL deterioration was also investigated.

The study followed the Declaration of Helsinki and the Guideline for Good Clinical Practice (ICH-GCP).

## Results

Baseline patient characteristics including age and sex were generally well balanced between treatment groups and have previously been reported elsewhere [5]. Disease characteristics were also similar between groups, including Karnofsky performance status, recurrence status, median time since first diagnosis, and tumour diameter [5].

**Fig. 1:**
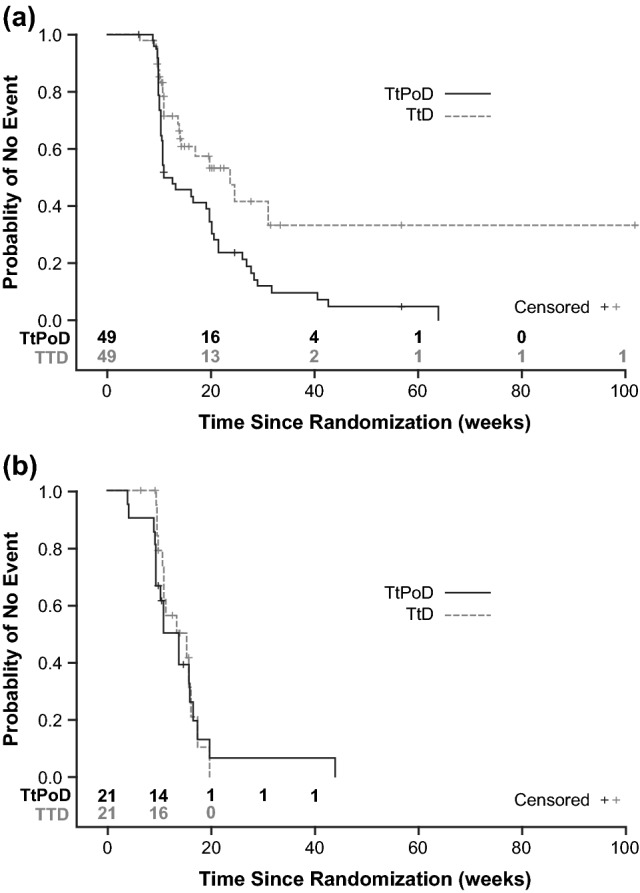
Kaplan–Meier curves showing TtPoD and TtD of overall QLQ-CL15 PAL in patients treated with Asunercept + rRT (**a**) or rRT alone (**b**)

**Fig. 2:**
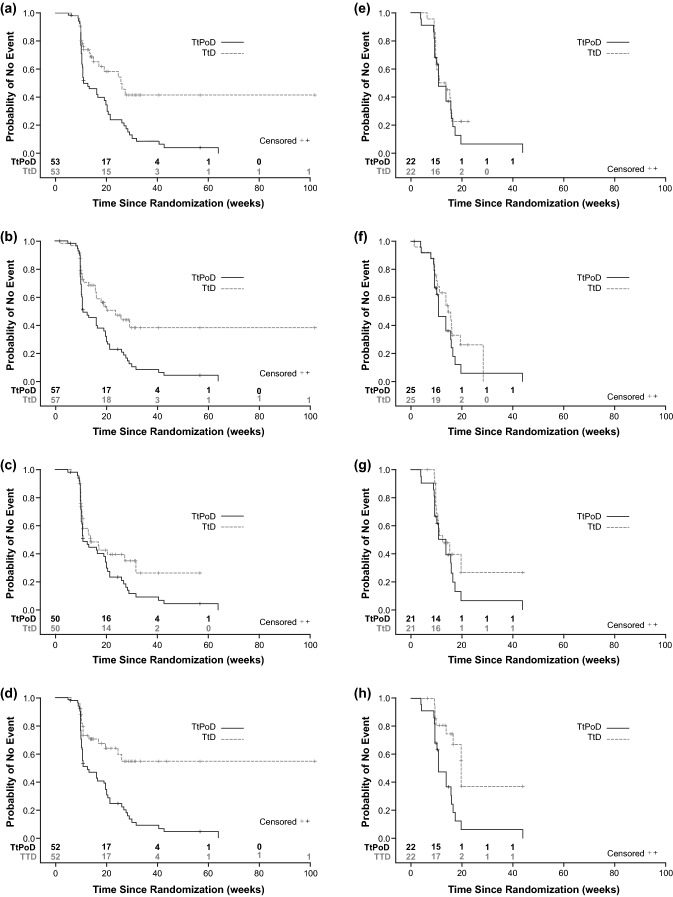
Kaplan–Meier curves showing effect of Asunercept + rRT and rRT alone on TtPoD and TtD in physical functioning (**a**, **e**) neurological status (**b**, **f**), fatigue (**c**, **g**) and total sum of all scores (**d**, **h**)

Treatment with Asunercept + rRT was associated with significant prolongation of TtD compared with rRT alone for QLQ-CL15 PAL overall QoL and physical functioning, and MRC Neurological Status (p ≤ 0.05, Table 1). With Asunercept + rRT, overall QoL was maintained until TtD at 166 days (vs. 107 days with rRT, pp = 0.0099), and physical functioning was maintained until TtD at 183 days (vs. 89 days with rRT, p = 0.0069). MRC neurological status with Asunercept + rRT was maintained until TtD at 166 days (vs. 103 days with rRT, p = 0.0319). In the Asunercept + rRT group, QoL was maintained beyond PoD, as the proportion of patients without deterioration of QoL was considerably greater than the proportion of progression-free patients (Fig. 1a). In the rRT group the two curves nearly overlay, indicating a dependency between progression and QoL deterioration (Fig. 1b) A prolonged effect of Asunercept + rRT on QoL beyond PoD was observed for all scores (Fig. 2a‒d). For fatigue and total sum of scores (Brain Cancer Module 20 [BN20]), similar effects were also observed for treatment with rRT alone (Fig. 2g, h, Table 1).

**Table 1:** QoL deterioration and median TtD following treatment with either rRT + Asunercept or rRT alone

	Asunercept + rRT		rRT		p value
	N	Median TtD, days	N	Median TtD, days	
**QLQ-CL15 PAL**					
Overall QoL	49	166	21	107	0.0099
Physical functioning	53	183	22	89	0.0069
Emotional functioning	50	NR	21	117	0.3002
Fatigue	50	98	21	88	0.5956
**QLQ-BN20 total score**	52	NR	22	139	0.5419
**MRC neurological status**	57	166	25	103	0.0319

*NR* not reached

## Discussion

Due to the limited number of available therapies with substantial impact on PFS and OS in rGB, the maintenance of QoL has emerged as an important endpoint to reduce morbidity, preserve neurologic functions, and sustain the capacity to perform daily activities [22]. Compared with rRT alone, treatment with a combination of Asunercept + rRT was associated with a significant prolongation of TtD and maintenance of QoL. Disease progression is seen as a key event driving QoL deterioration, and the median TtD was comparable with PFS in both treatment arms. However, in patients receiving Asunercept + rRT the TtD was prolonged beyond progression of the disease; this was not the case in patients treated with rRT alone. In none of the scores examined did treatment with Asunercept have a negative impact on patient performance/QoL.

In the current study, PFS-6 and OS for Asunercept + rRT were in line with Phase II/III studies of approved treatments for rGB [23‒25]. QoL is a key consideration in studies of rGB, and, as such, other studies of approved interventions have also assessed the relationship between TtPoD and QoL deterioration, as summarised in Table 2. A Phase III study in which patients with rGB were randomised to receive lomustine plus bevacizumab (N = 288) or lomustine alone (N = 149) reported no significant difference in TtD in QoL between groups when progression was not included as an event [26]. Nonetheless, deterioration-free survival was longer in the combination group than in the monotherapy group (12.4 weeks vs. 6.7 weeks; pp < 0.001), reflecting the difference in time to progression [26]. CABERET, a Phase II trial of bevacizumab and carboplatin (N = 122) in rGB, reported that decreases in health-related QoL generally occurred before disease progression [27]. Despite this, QoL domains considered relevant to symptoms of rGB improved in half of the patients who had symptoms at baseline [27]. There were no differences between patients receiving bevacizumab alone and those given carboplatin [27]. A Phase II study comparing temozolomide (N = 112) with procarbazine (N = 113) in patients with rGB showed that, regardless of the treatment, QoL was maintained at baseline levels prior to PoD but then decreased substantially at the time of PoD [24]. In light of these studies, it is of note that our Phase II study demonstrated that, in addition to improved PFS-6, Asunercept + rRT maintained QoL beyond PoD, particularly within the domains of general QoL, physical functioning and MRC neurological status. In the current study, approximately half of the patients in each arm received bevacizumab after disease progression, as per the investigator’s choice, with different doses and durations of its administration. Our study did not include a QoL assessment specifically on bevacizumab, and thus we can neither confirm nor exclude its impact on QoL. Available data from published studies on bevacizumab do not support either prolonged OS or QoL (Table 2). This suggests that further studies are needed to fully explore how bevacizumab affects QoL in rGB.

**Table 2:** Summary of quality of life results in Phase II/III studies in patients with recurrent GB receiving systemic treatments

Study	Study design	Treatments	QoL questionnaire reported	QoL results	Relationship between progression of disease and QoL deterioration
Brandes et al. [34]	Phase II randomised study	Patients randomised to lomustine plus bevacizumab (n = 61), or lomustine plus placebo (n = 62) until further disease progression, then patients continued bevacizumab or placebo with chemotherapy (investigator’s choice)	EORTC Quality of Life questionnaire (QLQ-C30) BN20	No differences between treatment groups in time to health-related QoL deterioration	Not reported in publication
Badruddoja et al. [35]	Phase II single-arm study	Patients received bevacizumab and temozolomide (n = 30)	Functional Assessment of Cancer Therapy-Brain (FACT-BR) Patient and/or family member completed FACT-BR at every clinic visit during the duration of the study	No significant differences in mean FACT-BR scores between cycles	FACT-BR scores were unchanged until just prior to tumour progression Once the patient had progressive disease, FACT-BR data were no longer collected
Field et al. [27]	Phase II randomised study	Patients randomised to bevacizumab plus carboplatin (N = 60), or bevacizumab alone (n = 62)	EORTC Quality of Life questionnaire (QLQ-C30) BN20 validated measurement tools EQ-5D health outcome measure	No differences between treatment groups	Decreases in health-related QoL generally occurred before disease progression Nonetheless, QoL domains considered relevant to symptoms of rGB improved in half of patients who had symptoms at baseline
Wick et al. [26]	Phase III randomised study	Patients randomised to lomustine plus bevacizumab (N = 288), or lomustine alone (N = 149)	EORTC Quality of Life Questionnaire-Core 30 (QLQ-C30) EORTC brain-cancer module (BN20) Evaluated at baseline and every 12 weeks	No significant differences between treatment groups, apart from a lower score for social functioning in the lomustine + bevacizumab group versus the lomustine group (P = 0.001)	No significant difference in TtD in QoL between groups when progression was not included as an event Deterioration-free survival was longer in the lomustine + bevacizumab group versus the lomustine group (12.4 weeks vs. 6.7 weeks; pp < 0.001)
Dirven et al. [36]	Phase II randomised study	Patients randomised to lomustine (n = 45), bevacizumab (n = 46), or lomustine plus bevacizumab (n = 47)	EORTC Quality of Life questionnaire (QLQ-C30) Brain module (QLQ-BN20) Measured at randomisation and every 6 weeks until disease progression	Health-related QoL remained stable in all treatment groups during the first three treatment cycles > 50% of patients showed stable or improved health-related QoL during their progression-free time, except for social functioning, irrespective of treatment group	Reduced health-related QoL was most pronounced at disease progression for all scales, other than social functioning, which deteriorated earlier in disease course
Kong et al. [37]	Phase II study of two patient cohorts	First cohort of patients received temozolomide 40 mg/m^2^ (n = 10). Second cohort of patients received temozolomide 50 mg/m^2^ (n = 28)	Physical and mental component scores from the short form-36 (SF-36) Measured at baseline, 3 months, and 6 months from the beginning of treatment	Follow-up SF-36 at 3 months showed a significant decrease in QoL score in physical health status in all patients No significant difference in mental health status	For patients responding to treatment, SF-36 at 3 months demonstrated no significant difference in physical and mental health status when compared with baseline
Wick et al. [23]	Phase III randomised study	Patients randomised to enzastaurin (n = 174), or lomustine (n = 92)	Functional Assessment of Cancer Therapy-Brain (FACT-BR) Completed before randomisation, every 3 weeks, and after discontinuation	No difference between treatment groups in time to deterioration of physical and functional well-being and symptoms	Deterioration in patient-reported outcomes was consistent with PFS
Khan et al. [38]	Phase II single-arm study	Patients received temozolomide (n = 35)	Functional Assessment of Cancer Therapy–Brain (FACT-BR) Completed at study entry and at the end of each cycle		Most patients maintained FACT-BR scores during both stable and progressive disease No significant post-progression deterioration in the FACT-BR scores
Brada et al. [39]	Phase II single-arm study	Patients received temozolomide (n = 138)	EORTC QLQ-C30 (+3) Brain Cancer Module 20 (BCM-20)		Health-related QoL responses were more common among the patients who responded to treatment than among those with progressive disease Most improvement was recorded in global health-related QoL and motor dysfunction scores
Yung et al. [24]	Phase II randomised study	Patients randomised to temozolomide (n = 112), or procarbazine (N = 113)	EORTC QLQ-C30 (+3) Brain Cancer Module 20 (BCM20) Questionnaires were completed on day 1 of cycle 1 and at every visit throughout the study	Across all domains, the proportions of patients achieving a health-related QoL response were consistently higher for temozolomide than for procarbazine	Regardless of treatment, QoL was maintained at baseline levels prior to progression of disease, but then decreased substantially at the time of disease progression
Meta-analysis to investigate the added prognostic value of HRQoL for OS and PFS in a large heterogeneous sample of glioma patients					
Coomans et al. 2019 [28]	Meta-analysis including data from previously published RCTs in glioma patients in which HRQoL was assessed	5217 patients with glioma	QLQ-C30 and QLQ-BN20 questionnaires		Better cognitive and role functioning and less motor dysfunction were associated with longer OS Better role and cognitive functioning, and less nausea and vomiting were associated with longer PFS

A meta-analysis using data from 15 RCTs including 5217 patients was performed to investigate the added prognostic value of heath-related QoL for OS and PFS in glioma patients [28]. The study reported that factors including better cognitive and role functioning and less motor dysfunction were independently associated with prolonged OS [28]. Factors including better role and cognitive functioning, and less nausea and vomiting were independently associated with prolonged PFS [28].

Regarding the impact of different radiation schedules on outcomes in patients with GB, a recent study [29] used data from the National Cancer Database to identify patients with GB who underwent surgical resection and external-beam radiation with chemotherapy. The findings showed that dose-escalated radiotherapy has decreased with time in GB patients in the US, as supported by clinical guidelines [30, 31]. The study did not identify differences in survival between patients receiving conventional doses, and those receiving higher doses (>66 Gy). A recent multiple linear regression analysis of publications from 1992 to 2016 investigated the relationship between re-irradiation and median OS [32]. Findings suggested that OS was highest after re-irradiation with single-fraction stereotactic radiosurgery, followed by hypofractionated stereotactic radiotherapy, and conventionally fractionated radiotherapy. Reporting of health-related QoL outcomes remains an unmet need in rGB trials – this was not reported in these studies.

There are limitations to comparing QoL results between different studies due to factors including use of different scales, relevance of the domains included in the scales to the patient population, and differences in timing for when QoL measures were made. Table 3 summarises three of the most frequently used scales: EORTC QLQ-C30, BN20, and Assessment of Cancer Therapy–Brain (FACT-BR). Our study utilised the EORTC QLQ-C15-PAL questionnaire, which is designed to assess QoL in palliative care cancer patients and includes certain domains and symptoms of EORTC QLQ-C30: overall QoL, physical, emotional and fatigue. As such, this scale was more suitable for the patient population included in the current study. The FACT-BR and BN20 questionnaires were designed specifically for patients with brain tumours. FACT-BR largely focuses on emotional and social functioning and as such may be more useful in patients with good functional status but who have emotional and social concerns [33]. Used with QLQ-C30 or QLQ-C15-PAL, BN20 may provide a broader evaluation of QoL in studies concerned with functional status that might affect QoL [33]. Nonetheless, both of these questionnaires, and others used in studies of QoL in recurrent GB, are valid, have been used extensively and provide reliable results. A further limitation of our, and all other published data so far, is the lack of health-related QoL-follow-up after progression and the number of completed follow-up visits, even in larger trials.

**Table 3:** Summary of the most-frequently administered QoL questionnaires in recurrent GB

Scale	EORTC QLQ-C30 [40]	Brain cancer module 20 (BCM20/BCN20) [41]	Functional assessment of Cancer Therapy–Brain (FACT-BR) [42]
**Objective**	To assess the QoL of patients with cancer	To be used in conjunction with the EORTC QLQ-C30 for measuring the health-related QoL in patients with brain cancer	To measure general QoL that reflects symptoms or problems associated with brain malignancies
**Scoring scale used**	Not at all: 1 A Little: 2 Quite a Bit: 3 Very Much: 4 Two global health status/QoL items are measured on a 7-point Likert scale (“very poor” to “excellent”)	Not at all: 1 A Little: 2 Quite a Bit: 3 Very Much: 4	Five-point Likert scale ranging from 0 "not at all" to 4 "very much"
**Interpretation of results**	Patient responses are scored on health-related QoL measurement scales according to standard algorithms All scales have a 0–100 range. Improvement in function is represented by an increase in score, whereas improvement in symptoms is represented by a decrease in score	Patient responses are scored on health-related QoL measurement scales according to standard algorithms All scales have a 0–100 range. Improvement in function is represented by an increase in score, whereas improvement in symptoms is represented by a decrease in score	Higher ratings suggest higher QoL. Items are totalled to produce a score for each subscore (physical well-being; social/family; emotional well-being; functional well-being; and concerns relevant to patients with brain tumours), plus an overall QoL score
**Domains covered**	Includes 30 questions that cover five aspects of functioning (physical, role, emotional, cognitive, social), eight symptoms (fatigue, pain, nausea/vomiting, dyspnoea, insomnia, appetite loss, constipation, diarrhoea), financial impact, and global health status/QoL	Includes 20 questions that cover future uncertainty, visual disorder, motor dysfunction, communication deficit, headache, seizures, drowsiness, hair loss, itching, difficulty with bladder control, and weakness of both legs	Includes 23 questions on aspects of physical well-being, social/family well-being, emotional well-being, functional well-being, and disease-specific concerns

## Conclusion

In patients with rGB, treatment with Asunercept + rRT significantly prolongs TtD and maintains QoL versus rRT alone.

## Data Availability

Data generated or analysed during and after this study are included in this and previously published articles.
